# Probiotics, Prebiotics and Synbiotics in Inflammatory Bowel Diseases

**DOI:** 10.3390/jcm10112466

**Published:** 2021-06-02

**Authors:** Katarzyna Akutko, Andrzej Stawarski

**Affiliations:** 2nd Department and Clinic of Paediatrics, Gastroenterology and Nutrition, Medical University of Wroclaw, M. Curie-Skłodowskiej St. 50/52, 50-369 Wrocław, Poland; andrzej.stawarski@umed.wroc.pl

**Keywords:** Crohn’s disease, ulcerative colitis, probiotic, prebiotic, synbiotic

## Abstract

Inflammatory bowel diseases (IBD), which include Crohn’s disease (CD) and ulcerative colitis (UC), are chronic inflammatory diseases of the digestive tract with periods of remission and relapses. The etiopathogenesis of IBD is multifactorial and has not been fully understood. Hence, only symptomatic treatment of these diseases is possible. The current pharmacological treatment has variable efficacy and is associated with the risk of significant side effects. Therefore, there is a constant need to search for new types of therapies with a high safety profile. Considering that the qualitative and quantitative profile of the gastrointestinal microbiome is often different in patients with IBD than in healthy individuals, there is a need for looking for therapies aimed at restoring intestinal microbiome homeostasis. Thus, the use of strictly defined probiotics, prebiotics and synbiotics may become an alternative form of IBD therapy. There is evidence that treatment with certain probiotic strains, e.g., VSL#3 and *Escherischia coli* Nissle 1917, is an effective form of therapy to induce remission in patients with mild to moderate UC. So far, the effectiveness of the use of probiotics, prebiotics and synbiotics in inducing or maintaining remission in patients with CD has not been confirmed. There are also reports of possible beneficial effects of fecal microbiota transplantation (FMT) on the course of IBD, especially UC. Further, well-planned studies on a large group of patients are needed to determine the role of specific probiotic strains, prebiotics, synbiotics and FMT in the treatment of IBD in adults and in children.

## 1. Introduction

Inflammatory bowel diseases (IBD), including ulcerative colitis (UC), Crohn’s disease (CD) and unclassified inflammatory bowel disease (IBD-U), are characterized by chronic inflammation of the gut mucosa [1]. It is known that the etiology of IBD is multifactorial and complicated, hence it is still not fully understood. Chronic inflammation of the intestinal mucosa is likely the result of multidirectional interactions between environmental, microbiological, genetic and immunological factors [2,3]. This results in an imbalance of the immune system within the gastrointestinal mucosa and the dominance of pro-inflammatory over anti-inflammatory processes [4,5,6,7]. The quantitative or qualitative change in the composition of the gut microbiome is one of the most important factors regulating the intestinal immune system, and thus may influence the development and course of IBD [4,5,6]. The gut microbiome of patients with IBD differs from that of healthy individuals. Among others, in patients with IBD with both exacerbation and remission, the presence of *Clostridiaceae* or adherence-invasive strains of *Escherischia coli* (AIEC) is found more often than in the general population [5,8,9,10,11,12]. The alteration of gut microbiome may be the result of primary inflammation, but also the cause of chronic inflammation of gut mucosa, therefore resulting in IBD [4,5,6,7]. It has been shown that the use of antibiotics and the associated dysbiosis are risk factors for the development of CD, but also that quantitative and qualitative changes in the microbiome may be present in humans with a documented genetic predisposition to develop IBD, but without clinical symptoms [5,7]. Currently, there is no causal therapy for IBD available, and the symptomatic treatment used has variable efficacy and often numerous limitations [4]. Regardless of whether changes of the gut microbiome are primary or secondary to IBD, its modification through diet, the use of antibiotics or probiotics provides a strong theoretical basis for conducting further, well-planned research about the use of therapy restoring the microbial balance in the gut as another element of IBD therapy [4,13,14].

## 2. Mechanisms of Action of Probiotics

The World Health Organization (WHO) defines probiotics as living microorganisms, when administered in adequate doses, results in a beneficial effect on the health of the host [15]. The mechanisms of action and effects of probiotics vary and depend on the strain and dose. Different probiotics interact with the host in different ways. Some show direct antimicrobial activity through the production of substances like lactic acid, hydroperoxides, bacteriocins or defensins. Others show non-immunological activity, such as competing with pathogenic microorganisms for nutrients, altering the intestinal pH, increasing mucus production, enhancing tissue repair processes or promoting the formation of tight connections, thereby reducing the permeability of the intestinal mucosa. Finally, probiotics can also modulate the immune response (production of proinflammatory cytokines, production of immunoglobulins) by the release of cell wall fragments or DNA in the intestinal lumen [4,6,16]. The results of clinical trials regarding the use of probiotics in patients with IBD are not providing a firm conclusion. Reports on the effectiveness of probiotics in inducing or maintaining remission in IBD are contradictory. This may result from the variety of bacterial species or strains used as a probiotic, but also from methodological differences between the studies [5].

## 3. Gut Microbiome

In the human gastrointestinal tract 1000–5000 species of microorganisms were identified, mainly bacteria (96%), but also viruses and fungi, which constitute the intestinal microflora [4,5,6,7]. The large intestine contains the highest amount of microorganisms in the human body. There are 10^12^ microorganism cells in 1 g of intestinal contents [13,17]. In healthy individuals, more than 90% of intestinal bacteria species belong to four main groups: *Bacteroides*, *Firmicutes*, *Actinobacteria* and *Proteobacteria* [7,13,17,18,19]. Microorganisms, through various mechanisms, directly or indirectly, affect the human immune, endocrine and nervous systems, and thus modify both local and systemic homeostasis [4,7,13,18]. Inter alia, *Bacteroides fragilis* stimulates the differentiation and activity of regulatory T cells, which are responsible for the inhibition of inflammatory processes. *Mucispirillum* species stimulate the secretion of Ig A, which is important in local defense mechanisms against non-symbiotic bacteria [4]. Some authors suggest that the effect of probiotics on inducing and maintaining remission in patients with IBD is dependent on their immunomodulatory effects [6]. The microorganisms of the intestinal flora can also influence the host organism indirectly through the products of its own metabolism or products resulting from intestinal bacterial fermentation. E.g., the produced short-chain fatty acids (SCFA) are necessary to maintain the integrity of the intestinal mucosa [4,17,18,19]. The composition of the intestinal microflora is variable and depends on genetic factors, diet or therapy [4,5,6,7]. Changes in composition of the intestinal microflora, in comparison to healthy individuals, may lead to the development and persistence of a number of diseases including IBD [4,18,20].

## 4. Gut Microbiome Changes in Patients with Inflammatory Bowel Diseases

Environmental factors causing dysbiosis have been reported to be one of the leading causes of IBD. However, what mechanisms triggered by dysbiosis ultimately lead to the development of chronic inflammation remains unclear [4,18]. Both quantitative and qualitative changes in the composition of the gut microbiome are found in patients with IBD. The most common difference is decline in bacterial species and genera, resulting in less biodiversity [5,13,20]. There is also a change in the proportion between different types of bacteria, e.g., the number of *Proteobacteria* is increased and the number of *Firmicutes* is reduced in the stool of patients with IBD compared to the control group [20,21,22]. Other studies have shown that in patients with IBD there is a reduction in the number of bacterial species with anti-inflammatory properties (e.g., *Faecalibacterium prausnitzii*) and an increase in the number of pro-inflammatory bacterial species (e.g., *E. coli*) [5,9,13,23]. Additionally, comparing patients with IBD in remission to patients with clinically active disease, differences in the qualitative and quantitative composition of the gut microbiome were shown. This indirectly proves a significant correlation between the change in the microbiome and chronic inflammation of the intestinal mucosa [5]. Some studies suggest that in patients with IBD there is a decrease in the number of aerobic bacteria and an increase in the number of anaerobes, which directly increases the permeability of the intestinal mucosa [4,24]. Other studies have shown a reduction in the number of obligate anaerobes [22]. This indicates that various disturbances in the composition of the gut microbiome may favor the development of IBD. It was also found that in patients with IBD, the number of non-toxigenic *Clostridium* spp., which induce TGF-β and IL-10 dependent T-regulatory activity, is significantly reduced, thus reducing inflammation and improving the intestinal barrier function. Therefore, reducing the amount of these bacteria reduces the potential to induce natural immunosuppressive processes necessary to maintain the balance of inflammatory reactions in the intestinal wall [4,19,25]. It has been reported that patients with CD have less variety of *Firmicutes* (especially fewer *F. prausnitzii*) and more *Enterobacteriaceae* (especially more *E. coli*) than healthy individuals [4,7,13]. Additionally, an increase in the amount of *Clostridium perfringens* in patients with UC compared to healthy individuals has been noticed. It has also been shown that patients with active UC have quantitatively less *Fusicatenibacter saccharivorans* than patients with UC in clinical remission [13].

Although fungi are only 0.02–0.03% of the intestinal microflora composition, they may also have a significant impact on the development of IBD [13,20,26]. The number of species and the variety of fungal types are higher in mucosal biopsies of patients with CD compared to the control group, which is the opposite of what was observed for bacteria [20]. It has been proven that species such as *Candida albicans* or *Saccharomyces boulardii* have the potential to modulate the inflammatory response of the intestinal mucosa [13,26]. Patients with IBD also showed an increased *Basidiomycota/Ascomycota* ratio, a decreased percentage of *Saccharomyces cerevisiae* and an increased percentage of *C. albicans* or *Cryptococcus neoformans* compared to the healthy individuals [20,26]. It has also been shown that in patients with IBD there is an increased ratio of the diversity of fungal species to bacteria, and studies in mice have shown that an increased amount of fungi in the intestinal microbiome is a predisposing factor for the development of UC [13].

## 5. Probiotics in Inflammatory Bowel Diseases

A systematic review by Langhorst et al. [27] shows that patients with IBD have a particular interest in the use of complementary and alternative medicines. This is often due to the fear of side effects or the lack of effectiveness of treatment with conventional drugs. Patients with IBD also show great interest in the possibility of treatment with probiotics. Research on the use of probiotics in the treatment of IBD has been conducted since 1997 [6]. Probiotics are often used by patients with IBD. Among patients with IBD even a 50% increase in the use of probiotics has been reported. This is due to the belief that probiotics are safe and have a beneficial effect as an additional form of therapy in patients with IBD, both during periods of exacerbation and remission [20,28]. Despite the relatively large number of reports on the use of probiotics in IBD, the possibility of unambiguous conclusions is significantly limited. This is due to the small number of patients in the study groups and significant differences in the types of intervention or the lack of standardization of research methods [4,13,20]. There are also only a few published clinical studies on the effects of probiotics on inflammatory changes assessed in gastrointestinal endoscopy in patients with IBD [5]. However, the potential use of well-selected, commensal species of microorganisms with a protective effect on the intestinal mucosa and modulation of immune responses gives hope for new therapeutic possibilities for patients with IBD [20,29]. In clinical trials, probiotics were used in therapy supporting the treatment of IBD, including the prevention of dysbiosis during long-term antibiotic therapy or immunosuppressive therapy, as well as in the treatment of dysbiosis in patients with newly diagnosed IBD or with exacerbation of the disease [4,17]. In recent years, an increase in the coexistence of symptoms of functional disorders of the gastrointestinal tract in patients with IBD with low clinical activity, measured by objective indicators (e.g., the concentration of calprotectin in the feces) has been observed. There are many reports of the effectiveness of probiotics in the treatment of functional abdominal pain, so the use of probiotics in the treatment of patients with overlapping functional disorders and IBD can bring significant benefits [5].

### 5.1. Probiotics in Ulcerative Colitis

The colon has the highest concentration of microbes in the human body. Thus, therapy to normalize abnormalities in the composition of the colon microbiome could theoretically bring significant benefits to patients with UC. Several probiotic strains have been studied and may have significant benefits for patients with UC [4,13]. The results of these tests are presented in Table 1 [30,31,32,33,34,35,36,37,38,39,40,41,42,43] It has been shown that the use of the non-pathogenic strain of *E. coli* Nissle 1917 had a similar efficacy and comparable safety profile in maintenance therapy as treatment with salicylates in patients with mild or moderate UC [4,13,32,44]. Similarly, Zocco et al. [36] showed that the supply of *Lactobacillus rhamnosus* GG as monotherapy or together with mesalazine in patients with UC significantly prolonged clinical remission during one-year follow-up compared to the group treated with anti-inflammatory mesalazine alone. The use of various lactic acid bacteria and *Bifidobacteria* as adjuvant therapy also significantly improved the course of the disease and the maintenance of clinical remission in patients with UC [4,29]. Several pilot studies have also shown that the nonpathogenic yeast *S. boulardii* has been successfully used for both induction and maintenance of remission in patients with mild to moderate UC [13,39]. Additionally, the use of rectal enemas containing *Lactobacillus reuteri* ATCC 55730 as adjunctive therapy to 5-aminosalicylic acid (5-ASA) in children and adolescents with mild or moderate distal UC resulted in endoscopic and histopathological improvement compared to the placebo group [40]. One of the best-studied is probiotic complex VSL#3 consisting of four *Lactobacillus* strains (*L. casei*, *L. plantarum*, *L. acidophilus* and *L. delbrueckii* subsp. *Bulgaricus*), three *Bifidobacterium* (*B. longum*, *B. breve* and *B. infantis*) and one *Streptococcus* (*S. salivarius* subsp. *Thermophilus*) [4,13,29,45]. In studies carried out, so far on mouse models, it has been shown that the use of this probiotic mixture leads to the inhibition of NF-κB and TNF-α expression through the TLR4-NF-κB signaling pathway. As a result, the expression of pro-inflammatory cytokines and toll-like receptors (TLR) is reduced, and the level of regulatory cytokines increases [45,46]. It has been shown that the use of VSL#3, both as adjuvant therapy and in monotherapy, has been effective in inducing and maintaining remission in patients with mild to moderate UC [4,13,43]. Tursi et al. [41] also reported that the combined use of the standard therapy and VSL#3 in inducing remission in these patients is more effective than the standard therapy alone. A systematic review and meta-analysis by Derwa et al. [5] from 2017 confirmed that the VSL#3 probiotic mixture may have a beneficial effect on inducing remission in patients with UC and is as effective in preventing exacerbations as 5-ASA. In other studies, Miele et al. [43] showed that the use of VSL#3 as an adjunct to standard therapy is effective in inducing and maintaining remission in children with newly diagnosed UC compared with patients receiving placebo during one year of follow-up. The use of VSL#3 in the treatment of patients with UC is particularly important in the presence of 5-ASA intolerance [29]. However, a meta-analysis using rigorous statistical methods showed that the efficacy of VSL#3 and *E. coli* Nissle 1917 in treating exacerbations as well as maintaining remission in patients with UC is limited. The results are inconclusive, indicating the need for further research to finally determine whether the use of these probiotics in the treatment of UC is beneficial [4,13]. Moreover, it should be noted that the quantitative composition of the VSL#3 probiotic mixture has changed recently, and although it contains the same bacteria, its effectiveness in the treatment of patients with IBD has not yet been proven in clinical trials [43,44]. It should also be emphasized that the use of probiotics, like other drugs, is associated with the possibility of significant side effects. There are reports on both children and adults that the supply of probiotics, e.g., *L. rhamnosus* GG, may cause the development of bacteremia and sepsis in patients with reduced immunity or acute severe colitis [47]. Therefore, in the treatment of IBD, only specific probiotic strains with documented beneficial effects on the course of mild or moderate inflammatory disease should be used [44]. According to the recommendations of the European Society for Clinical Nutrition and Metabolism (ESPEN), the use of certain probiotics (VSL#3, *E. coli* Nissle 1917) may be considered in the treatment inducing remission in patients with mild or moderate UC. Probiotics should not be used in severe UC [44,48]. The use of probiotics as an alternative therapy may be particularly useful in treating patients with 5-ASA intolerance [44,49].

### 5.2. Probiotics in Crohn’s Disease

Data on the use of probiotics to induce or maintain remission in patients with CD are limited [5,13]. Therefore, the ability to analyze, compare the results and infer on this basis is unreliable [5]. Several studies aimed at the evaluation of efficacy of different probiotic strains both in inducing and maintaining remission, as well as in prevention of exacerbations after surgery in patients with CD (Table 2) [50,51,52,53,54,55,56,57,58]. The use of probiotics has not been shown to be associated with a clinically significant benefit for patients with CD either in single studies or in a meta-analysis [4,13,59]. *L. rhamnosus* GG did not benefit children with CD better than placebo. Moreover, the meta-analysis showed that the use of *L. rhamnosus* GG may even increase the frequency of relapses in children with CD [49]. VSL#3 was also not effective in the treatment of this group of patients [4,49]. It has been shown both that VSL# 3 as adjuvant therapy to 5-ASA reduced the frequency of relapses, but also that such enhancement of treatment did not bring any significant benefit to patients with CD [4,46,49]. Initial reports of Plein et al. [55] indicated that the use of probiotic strains of yeast *S. boulardii* reduced the frequency of exacerbations in adult patients with CD, but this was not confirmed in later studies [54]. A systematic review has shown that at least 120 well-designed, high-quality studies are necessary to clearly assess the impact of probiotic treatment on the course of CD [13]. Currently, there is no evidence that the use of probiotics is beneficial for maintaining remission in CD [59]. The current treatment guidelines for children and adults with CD published by ESPEN, the European Crohn’s and Colitis Organization (ECCO) and ESPGHAN are consistent [60]. Taking into account that so far no statistically significant benefits of using probiotics to induce or maintain remission compared to standard therapy have been found, probiotics should not be used in the treatment of patients with CD [44,59,60].

## 6. Prebiotics in Inflammatory Bowel Diseases

Prebiotics are fermentable carbohydrates with a wide variety of chemical structures that are administered for local or systemic health benefits [61,62,63]. Prebiotics may change the composition of the intestinal microbiota, improve the function of the intestinal barrier, and stimulate microbes of the digestive tract to produce metabolites beneficial for the host [63,64,65]. As in the case of probiotics, it is also very difficult to conduct clinical trials allowing for clear conclusions regarding the use of prebiotics in specific disease entities. Therefore, data on the use of prebiotics in patients with IBD are limited [64]. Hypotheses about the possibility of using prebiotics as a part of IBD therapy assume that supplementation with selected fiber fractions, including fermenting carbohydrates, aimed at the promotion of specific bacteria and/or the production of specific metabolites by specific bacteria, may cause the assumed beneficial effect for the host [63,64]. It seems that the use of prebiotics may be useful especially in patients with low clinical activity of the disease or to maintain remission. Most prebiotics used in studies of patients with IBD are classes of oligosaccharides and inulin [64]. In animal studies, it was shown that the administration of prebiotic fructans and resveratrol increased the amount of *Bifidobactrium* and *Lactobacillus* in the colon in IBD-induced mice and rats [65,66]. In vitro studies have shown that slow fermenting microspheres trapped in starch can induce beneficial changes in the profile of colon bacteria in patients with IBD by producing large amounts of butyrate, keeping the pH of the distal colon low and inhibiting the growth of potentially harmful bacteria [61]. Additionally, the oral supply of inulin to rats with induced chronic enteritis resulted in a reduction in the severity of lesions in the colon and had a positive effect on the profile of intestinal bacteria by increasing the amount of *Lactobacillus* and lowering the pH in the large intestine [67]. Studies in humans with IBD showed that the use of psyllium husk alleviated gastrointestinal symptoms in patients with UC in remission [68]. Casellas et al. [69] compared mesalazine therapy in combination with oligofructose-enriched inulin and placebo in patients with mild to moderate UC. Oral oligofructose-enriched inulin was well tolerated, and its supply resulted in a significantly earlier decrease in fecal calprotectin. Several clinical trials have been conducted in Japan to treat UC with germinated barley food (GBF), products which are mainly composed of dietary fiber and glutamine-rich protein. It has been shown that GBF may reduce clinical activity in patients with mild to moderate UC and appears to be an effective therapy for maintenance remission in these patients. It is important that such treatment seems to have a high safety profile, as the GBF used was not associated with the occurrence of any side effects in the study group [70,71]. However, in a double-blind, randomized, placebo-controlled trial Benjamin et al. [72] showed that the use of fructose-oligosaccharide (FOS) was not associated with any health benefit for patients with CD.

## 7. Synbiotics in Inflammatory Bowel Diseases

Synbiotics are products that contain both probiotics and prebiotics [65,73]. The term synbiotic refers to synergism, so it should be reserved only for products in which the prebiotic compound selectively favors the probiotic organism. Synbiotics were developed to overcome the potential difficulties of probiotics in survival, especially while passing through the upper gastrointestinal tract. The use of a synbiotic is therefore to contribute to a more effective implantation of a probiotic in the colon and to promote the growth of probiotic strains [63]. In the literature there are single reports on the beneficial effect of synbiotics on the course of IBD [64]. In a randomized, double-blind, placebo-controlled trial in 18 patients with UC, Furrie et al. [74] found that the use of a synbiotic consisting of *B. longum* and oligofructose-enriched inulin helps to reduce both macroscopic lesions assessed in sigmoidoscopy and microskopic inflammatory lesions assessed during histopathological examination of the rectal mucosa biopsy, while reducing the level of pro-inflammatory cytokines, like TNF-α and IL-1β. Those findings have not been confirmed by Hansen et al. [75] in a study among children with IBD. In a prospective multicenter, randomized study Chermesh et al. [76] showed that treatment with a mixture of four probiotic species and four prebiotics had no beneficial effect on postoperative recurrence in 30 enrolled patients with CD. Combinations of synbiotics can have positive effects on the intestinal mucosa. Thus, the assessment of the role of synbiotics as an alternative form of IBD therapy should be investigated [65].

## 8. Prebiotics and Synbiotics in Inflammatory Bowel Diseases—Recommendations

Currently, there is no evidence that the use of prebiotics or synbiotics could beneficially modify the course of IBD. The value of the studies conducted so far is limited (small numbers of the study groups), and their results are difficult to compare (short duration, high dropout rates, methodological differences). There is also little data on the influence of prebiotics and synbiotics on the course of IBD in children. Therefore, the use of prebiotics and/or synbiotics in inducing and maintaining remission of IBD in both adults and children is actually not recommended [49].

## 9. Fecal Microbiota Transplantation in Inflammatory Bowel Diseases

Fecal microbiota transplantation (FMT) involves introducing feces from a well-tested, healthy donor into the gastrointestinal tract of a person with a specific chronic disease in order to restore the normal intestinal microbiome and relieve the patient from pathological symptoms. Currently FMT is most popular for treatment of recurrent *Clostridioides difficile* infections [77]. It has been shown that in patients with IBD treated with FMT with a good outcome, an increase in colon microbiota diversity was noticed. In FMT recipients, the composition of the microbiome tended to shift towards the donor profile [78]. Dysbiosis is an important part of the multidirectional abnormalities observed in patients with IBD. FMT, like probiotics, as an element of therapy correcting dysbiosis provides theoretical grounds for conducting clinical trials on the use of FMT in patients with IBD [77]. There are reports that the use of FMT in patients with UC resulted in long-term clinical remission, which necessitates further research in this field. Costello et al. [79] presented a case of a 19-year-old man treated in the past with mesalazine, azathioprine and infliximab, currently with a severe UC relapse, who received FMT with a very good effect—the patient was in clinical and endoscopic remission 8 weeks after FMT. Additionally, after 12 months of follow-up, the patient was in clinical and endoscopic remission of UC. Tian et al. [80] conducted a prospective, uncontrolled placebo study of FMT in 20 patients with UC and showed that such treatment can significantly alleviate clinical symptoms and partially reduce colon mucosal lesion. Gutin et al. [81] conducted a prospective, open-label study which enrolled 10 patients with CD who received FMT once. Given that 3/10 patients responded to FMT but 2/10 patients had significant side effects, it was found that one-time FMT in this group of patients was associated with a reduced probability of improvement and the possibility of worsening the course of IBD. Xiang et al. [82] assessed the efficacy of FMT in 174 patients with CD. The authors showed that most of patients achieved improvement in clinical symptoms: diarrhea, abdominal pain, hematechezia and fever. Additionally, 50% of steroid-dependent patients with CD achieved steroid-free remission. It is also important that 24 studies analyzing the composition of the microbiota of FMT recipients showed a favorable change in the qualitative and quantitative profile of the colon microbiome and the pattern of colonic microbiota diversity. FMT shows some efficacy in inducing remission in patients with UC, but the long-term effect and safety have not been conclusively assessed [83]. Those observations indicate the need of further research to explain the role and assess efficacy of FMT as a part of IBD treatment.

## 10. Conclusions

The gut microflora is the center of a very diverse and complex human microbiome. Undoubtedly, qualitative and quantitative disorders of its composition in genetically predisposed persons may contribute to the development of IBD. Probiotic bacteria and fungi can significantly modulate the pro-inflammatory and anti-inflammatory pathways. Additionally, FMT can significantly change the qualitative and quantitative composition of the gut microbiome, and therefore it can modulate the local inflammatory response in the colon. Understanding the mechanisms of action of specific probiotics can be used to develop new therapeutic options for IBD based on selected bacterial strains or mixtures composed of several synergistically acting strains of bacteria, fungi and/or prebiotics. A better understanding of the mechanisms responsible for the effectiveness of FMT may allow for the identification of criteria for selecting an appropriate stool donor for the individual needs of the patient. However, it is necessary to determine the optimal doses, method of administration and duration of those therapies. Most of the clinical trials conducted so far have had significant limitations (including small numbers of study groups, high dropout rates, lack of appropriate efficacy analyzes, concomitant use of other disease-modifying drugs), making it impossible to reliably draw conclusions and use probiotics, prebiotics, synbiotics and FMT in the treatment of IBD. However, the existing reports of possible therapeutic benefits of the controlled modification of the gut microbiome in the treatment of IBD require further well-designed randomized controlled trials involving large patient populations to finally clarify whether the use of probiotics, prebiotics, synbiotics or FMT may beneficially modify the course of IBD and should be one of the therapeutic options for these diseases.

## Figures and Tables

**Table 1:** Results of clinical studies of using probiotics in ulcerative colitis patients.

Probiotic Used	Study	Sample Size	Studied Group	Result of the Intervention
*Escherichia coli* Nissle 1917	Kruis W. et al. Aliment. Pharmacol. Ther. 1997 [30]	120	adults	efficacy in maintaining remission and preventing relapse comparable to mesalazine
	Rembacken BJ. et al., Lancet. 1999 [31]	116	adults	efficacy in maintaining remission after exacerbation of UC comparable to mesalazine
	Kruis W. et al. Gut. 2004 [32]	327	adults	efficacy and safety in maintaining remission comparable to mesalazine
	Henker J. et al. Zeitschrift Für Gastroenterologie, 2008 [33]	34	children	efficacy in maintaining remission comparable to mesalazine
	Matthes H. et al. BMC Complement Altern Med. 2010 [34]	90	adults	possibility of dose-dependent efficacy in inducing remission of the rectal probiotic compared to placebo
	Petersen AM et al. J Crohns Colitis. 2014 [35]	100	adults	no benefit in the use of probiotic as an additional therapy to conventional treatment
*Lactobacillus* GG	Zocco MA, Aliment Pharmacol Ther. 2006 [36]	187	adults	higher efficacy of probiotic as add-on therapy in prolonging the relapse-free time compared to mesalazin monotherapy
*Bifidobacterium breve, Bifidobacterium bifidum, Lactobacillus* acidophilus YIT 0168 (Bifidobacteria-Fermented Milk- BFM)	Ishikawa et al. J Am Coll Nutr 2003 [37]	21	adults	higher efficacy of probiotic mixture as add-on therapy in maintaining remission and preventing relapse compared to convantional therapy alone
	Kato K. et al. Aliment. Pharmacol. Ther. 2004 [38]	20	adults	higher efficacy of probiotic as add-on therapy in maintaining remission compared to convantional therapy alone
*Saccharomyces boulardii*	Guslandi M. et al. Eur J Gastroenterol Hepatol. 2003 [39]	24	adults	higher efficacy of probiotic as add-on therapy in inducing and maintaining remission compared to mesalazin monotherapy
*Lactobacillus reuteri* ATCC 55730	Oliva S. et al. Aliment Pharmacol Ther. 2012 [40]	40	children	higher efficacy of probiotic enema as add-on therapy additional to oral mesalazin in improving mucosal inflammation compared to conventional therapy
*Lactobacillus casei*, *Lactobacillus plantarum*, *Lactobacillus acidophilus and Lactobacillus delbrueckii* subsp. *Bulgaricus, Bifidobacterium longum, Bifidobacterium breve and Bifidobacterium infantis, Streptococcus salivarius* subsp. *Thermophils* (VSL#3)	Tursi A. et al. Am J Gastroenterol. 2010 [41]	144	adults	higher efficacy of probiotic mixture as add-on therapy to conventional treatment in patients with relapsing disease compared to placebo
	Sood A. et al. Clinical Gastroenterology and Hepatology 2009 [42]	147	adults	higher efficacy in inducing and maintaining remission compared to placebo
	Miele E.et al. Am J Gastroenterol. 2009 [43]	29	children	higher efficacy in maintaining remission compared to placebo

**Table 2:** Results of clinical studies of using probiotics in Crohn’s disease patients.

Probiotic Used	Study	Sample Size	Studied Group	Result of the Intervention
*Escherichia coli* Nissle 1917	Malchow HA et al. J. Clin. Gastroenterol 1997 [51]	28	adults	higher efficacy of probiotic as add-on therapy in preventing relapse and reducing the need for steroid treatment compared to convantional therapy alone
*Lactobacillus* GG	Schultz M. et al. BMC Gastroenterology 2004 [52]	11	adults	no benefit in the use of probiotic as an additional therapy to conventional treatment
	Gupta P. et al. JPGN 2000 [53]	4	children	higher efficacy of probiotic as an add-on therapy in improving gut barrier function and clinical status
	Prantera C. et al. Gut 2000 [54]	45	adults	no benefit in preventing endoscopic relapses or reducing the severity of inflammation
	Bousvaros A. et al. Inflamm Bowel Dis. 2005 [55]	75	children	no benefit in use probiotic as add-on therapy to conventional treatment in prolonging of relapse- free time
*Saccharomyces boulardii*	Plein K. et al. Gastroenterol. 1993 [56]	20	adults	higher efficacy of probiotic as an add-on therapy in reducing in the number of stools compared to placebo
	Bourreille A. et al. Clin. Gastroenterol. Hepatol. 2013 [57]	165	adults	no benefit in maintaining remission as add-on therapy after conventional treatment
*Lactobacillus casei, Lactobacillus plantarum, Lactobacillus acidophilus and Lactobacillus delbrueckii* subsp. *Bulgaricus, Bifidobacterium longum, Bifidobacterium breve and Bifidobacterium infantis, Streptococcus salivarius* subsp. *Thermophils* (VSL#3)	Day AS. et al. Gastroenterology 2012 [58]	17	children	higher efficacy in reducing disease activity and improving weight and albumin levels compared do placebo
	Fedorak RN. Et al. Clinical Gastroenterology and Hepatology. 2014 [59]	120	children over 16 years old, adults	no benefits in reducing endoscopic recurrence rates compared to placebo
